# Geographical variability in morphology and nutritional composition of *Moringa oleifera* seeds: a meta-analysis

**DOI:** 10.3389/fpls.2026.1720005

**Published:** 2026-02-09

**Authors:** Preeti Sharma, Jayanti Tokas, Axay Bhuker, Baldev Raj Kamboj, Anurag Malik, Craig Robert McGill, Ajay Kumar Bhardwaj

**Affiliations:** 1 Department of Biochemistry, Chaudhary Charan Singh (CCS) Haryana Agricultural University, Hisar, Haryana, India; 2 Indian Council of Agriculture Research (ICAR)- Central Soil Salinity Research Institute, Karnal, Haryana, India; 3 Department of Seed Science & Technology, Chaudhary Charan Singh (CCS) Haryana Agricultural University, Hisar, Haryana, India; 4 Department of Agronomy, Chaudhary Charan Singh (CCS) Haryana Agricultural University, Hisar, Haryana, India; 5 Division of Research and Innovation, Uttaranchal University, Dehradun, Uttarakhand, India; 6 School of Agriculture and the Environment, Massey University, Palmerston North, New Zealand

**Keywords:** climatic zone, crude protein, moringa seed, morphological variability, nutritional composition, carbon-nitrogen metabolism

## Abstract

The miracle tree, *M. oleifera*, is valued for its nutritional composition, climate adaptability, industrial and environmental usefulness. Despite well-known benefits, nutritional composition varies with the geographical location. The seeds of Moringa are rich in high-quality oil and protein and are also a source of carbohydrates, but their relative quantities vary among geographical locations. A meta-regression analysis was carried out using PRISMA guidelines, to explore the variability, and deciding factors in Moringa seeds. A systematic search of Scopus and google Scholar identified reports that mentioned morphological or nutritional or both traits was carried out. After removing duplicates and reviews, total 31 original research articles were included in the study. Two independent datasets, morphological and nutritional, were prepared by extracting numerical data of mature seeds. Statistical framework included Pearson’s correlation to quantify trait relationship and ANCOVA to assess covariate effects on nutritional components. Datasets were analyzed using R software. Random effect meta regression model was employed to assess the heterogeneity in nutrient composition across climatic zones. The crude fat, total carbohydrates and crude protein were highly variable (σ = 14.56, 14.54 and 12.08 respectively). The variabilities in ash and moisture were low (σ =1.41 and 2.48 respectively) while crude fiber showed intermediate variability (σ = 2.87). Although, there was a trend in nutritional composition of *M. oleifera* seeds along the latitude and climatic zones, statistical model fitting was non-significant for these variables. Pearson’s correlation among nutritional components was pronounced and significant, supported by carbon-nitrogen metabolism. This study did not find any trend in the highly variable morphological components (CV 38.52% and 43.12% for length and width respectively) of Moringa seeds with geographical location.

## 1 Introduction

Moringa (*Moringa oleifera*), often referred to as the “miracle tree,” is of significant importance owing to its exceptional nutritional and medicinal properties. This fast-growing plant is native to parts of Africa and Asia, and has gained global recognition for its multifaceted benefits. Moringa leaves, seeds, and pods are rich in essential vitamins, minerals, and antioxidants, making them a valuable source of nutrition, particularly in regions facing food insecurity (Gopalkrishnan et al., 2016; Pandey et al., 2019; Rai et al., 2023). The plant’s medicinal properties have been utilized in traditional medicine for centuries, with potential applications in treating various ailments, including inflammation, diabetes, and cardiovascular issues. Additionally, moringa’s industrial potential, ability to purify water, and its use as a sustainable crop for both human consumption and livestock feed further underscore its importance in addressing global challenges related to nutrition, health, and environmental sustainability (Mulugeta and Fekadu, 2014).

In the past decade, moringa has gained the attention of researchers and they are trying to explore the medicinal, agricultural, environmental, and industrial potential of the crop. Like other parts of the plant, moringa seeds are a rich source of nutrients. These are particularly considered a good source of high-quality oil and protein. Moringa seed oil is extensively used in cosmetic industry, has the potential to be used as edible cooking oil and can be used to produce biodiesel. Moringa seed proteins are unique in their ability purify water. But high variations in moringa seed oil and protein content are evident from the scientific research. Banerji and Bajpai (2009) reported that oil content of moringa seeds from 20 clones of India was higher than the oil content reported from Pakistan, Malaysia and Kenya. Other crops such as soyabean and barley also exhibit differences in nutrient composition of seeds when grown in different environmental conditions (Bellaloui et al., 2016; Xue et al., 2016). According to Rani et al. (2025), environmental conditions play important role in determining quality of moringa seeds. An analysis of variation in nutritional composition of seeds across various agroclimatic regions could help make informed decision on the sourcing of seeds for particular application.

While, influence of location is evident, none of the study comprehensively covers worldwide locations. The current data on seed morphology and nutritional composition of moringa seeds is highly fragmented and variable with individual studies reporting inconsistent estimates due to differences in geographical origin, climatic conditions, genotypes, and analytical methodologies. Meta-analysis is essential in this study to integrate widely scattered data, generate more precise estimates of trait variability, and evaluate the influence of geographical and climatic factors on Moringa seed quality, which cannot be achieved through individual studies alone.

Apart from variations in basic nutritional components, various secondary metabolites also accumulate differentially. Accumulation of phenols, tannins, flavonoids, terpenes, etc. is higher in adverse environmental conditions (Qaderi et al., 2023). Environmental stressors act as stimuli that change gene expression pattern and therefor alter biochemical pathways. Secondary metabolites being protective in nature, accumulate to enhance plant adaptability to stresses and alter the quality of seed. Secondary metabolites are derived from primary metabolites. Studying variations in proximate values of primary metabolites will provide a stepping stone in further investigations of these specialized compounds.

This study covers locations across globe which were not uniformly dispersed but concentrated near equator in tropical and subtropical zone. There were no reports from temperate region. Moreover, the weather conditions, seasons and accurate location of sampling were not uniformly presented in the selected reports, which may be critical in determining the quality of seeds. Therefore, by considering approximate coordinates, we presented a broader view of trends and correlations in various traits. The purpose of this study is to explore the factors affecting morphological variability and nutritional composition of moringa seeds across various agroclimatic regions through meta-analytic approach.

## 2 Materials and methods

**Figure 1:**
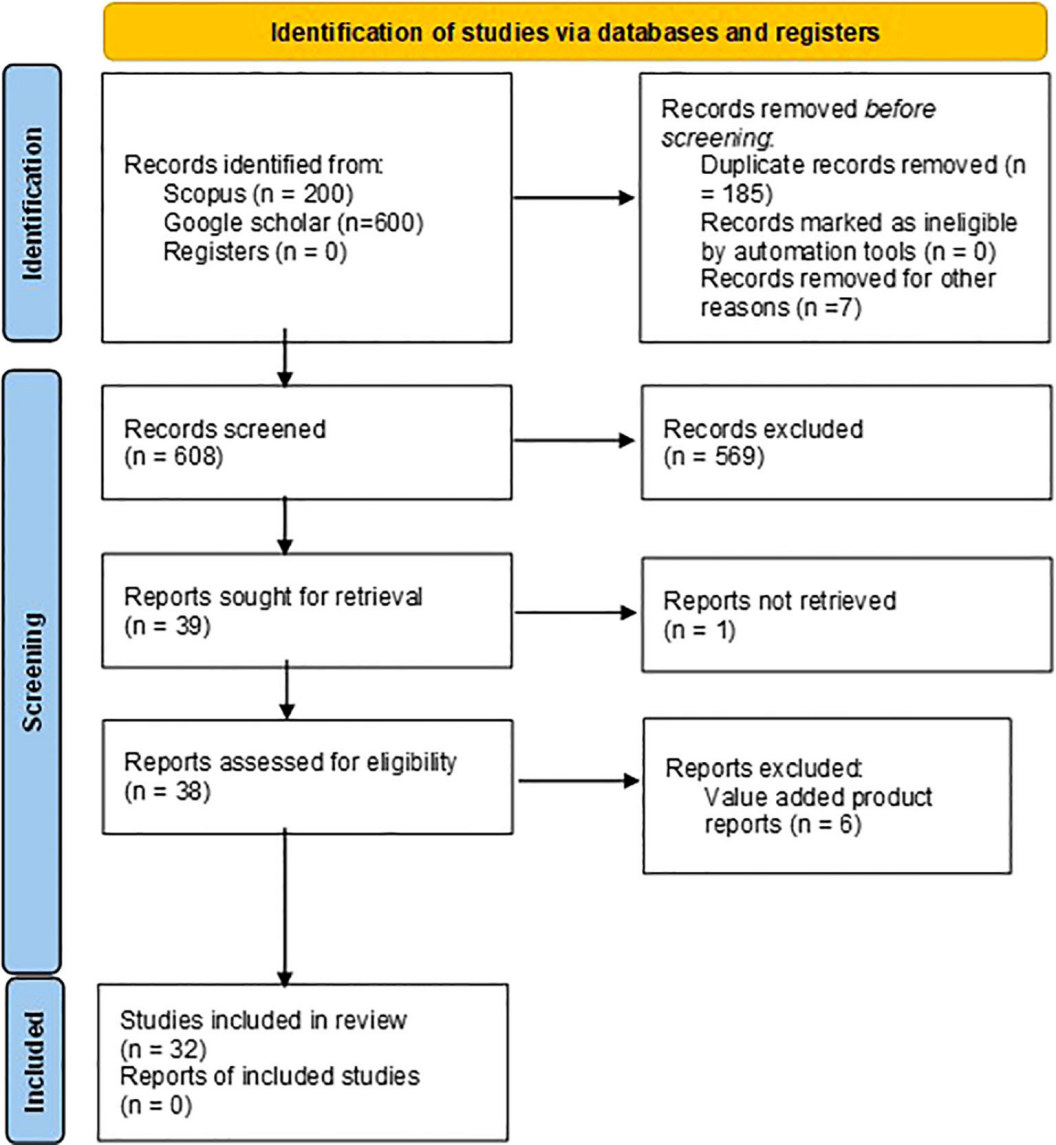
PRISMA flowchart for screening and selection of literature from databases.

The keywords *“Moringa oleifera* seed morphology”, “*Moringa oleifera* seed proximate composition” and “*Moringa oleifera* seed nutritional composition” were used to search relevant research articles in Scopus database and Google Scholar. A total of 200 studies were identified with the relevant keywords in the title and abstract in Scopus. Publish or perish software was used to extract top 200 studied from google scholar for each keyword search. A total of 600 studies were extracted from Google scholar. All the 800 studies were imported in Zotero reference manager and 185 duplicated were removed. 3 irrelevant erratum and 4 conference abstracts were removed. 608 articles were screened manually for morphological and nutritional composition related work on raw moringa seeds and total 569 article were excluded. 39 articles sought for retrieval where 1 article was not retrieved. 38 reports were assessed for eligibility and 7 found ineligible because raw seed morphological or nutritional data or was not found in the articles (nutritional data of value-added product was given). A total of 31 studies included in the review. The study selection process is summarized in the PRISMA 2020 flow diagram (**Figure 1**). Required data was extracted and two datasets were prepared: morphological and nutritional composition.

The analysis was performed irrespective of the age (full mature fresh, full mature dry or non-mature) of the seeds. Only datasets of moringa seeds grown without any stressor or treatment were selected. Numerical data for morphological and nutritional traits were directly extracted from tables and figures presented in the included studies using a predefined extraction sheet. Because extraction involved only objective quantitative values rather than subjective interpretation or coding, inter-rater reliability measures were not applicable. Data accuracy was ensured through independent cross-checking of all entries by a second reviewer.

The locations were geocoded utilizing *tidygeocoder* package in R, which uses the OpenStreetMap Nominatim API (Cambon et al., 2021). Where exact location was not known, geocoding returned the centroid of the nearest identifiable administrative region.

**Figure 2:**
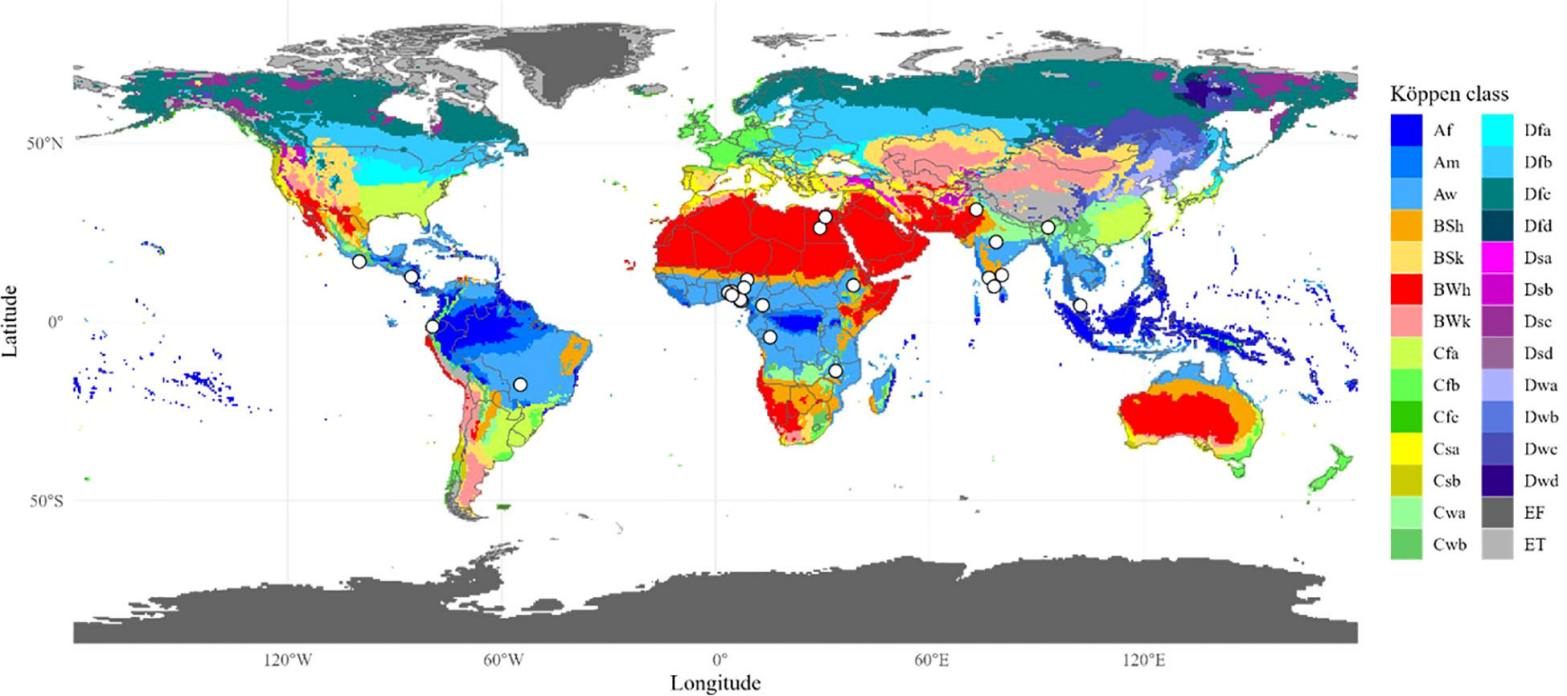
Koppen-Geiger climate zones with study locations. Koppen-Geiger climate map is widely accepted. The white dots indicate study locations. These dots were visually inspected for the respective climatic zone. Full names of abbreviated climatic zones are provided in supplementary file.

Koppen-Geiger climate map (Beck et al., 2023) was loaded as a raster in R (Lemenkova, 2020) with a resolution of 0.5° and geocoded locations were spatially overlaid on this raster to extract the corresponding climatic zone and generate the climatic map (**Figure 2**). Broad climatic zones were assigned to the study locations.

### 2.1 Morphological characteristics of *M. oleifera* seeds

A total of eight studies covering 59 accessions of 9 countries were selected to compare moringa seeds for morphological characteristics (**Table 1**). The seed shape, color, test weight, length, width, and wing color characteristics were considered. Data is harmonized by unit conversion to gram for test weight and centimeter for length and width. Based on the data, a qualitative estimate of the seed shape, seed color, and wing color parameters was performed manually. A lack of quantitative data pertaining to these traits in the selected studies restricted the quantitative estimate. The statistical analysis of the quantitative traits (weight, length and width) was then performed.

**Table 1:** Studies on the morphological characteristics of *M. oleifera* seeds.

Country	No. of accessions	Seed shape	Coat colour	Test weight (g)	Size (cm)		Wings colour	References
					Length	Width		
Mexico	22	Oval, round, triangular	Light brown to dark brown	21.29-38.60	0.98-1.54	0.74-1.11	Brown	Ruiz-Hernández et al., 2021
Nigeria (Ilorin)	30	Ovate, isodiametric	Tan, cream	–	1.02-1.50	0.81-1.11	–	Zhigila et al., 2015
Pakistan	3	globular	Pale yellow to whitish	–	–	–	Pale yellow to whitish	Anwar and Rashid, 2007
Egypt	1	Globose or sub-globose	Brown	–	2.5–2.7	2–2.6	–	Hamada et al., 2024
São Paulo, Brazil	1	Globose	Medium brown	19.4-20.0	0.714-1.037	0.807-1.195	Light brown	Ramos et al., 2010
Macaíba, Brazil	1	Globose	Brown	18.6-29.27	1.31-1.89	–	–	Noronha et al., 2018
Cameroon	1	–	–	31.6 ± 0.6	1.31 ± 0.23	1.14 ± 0.21	–	Saa et al., 2022
Ecuador	1	–	–	27	0.917	1.044	–	Coello et al., 2020
Republic of Benin (Africa)	29	–	–	20	0.87-1.89	–	–	Gandji et al., 2020
Morocco	39	–	–	1.14-6.23	–	–	–	Labbassi et al., 2024

‘-’ Indicate that data is unavailable.

### 2.2 Nutritional composition of *M. oleifera* seeds

A total of 23 studies covering 12 countries with 27 locations were selected for the study of proximate composition (**Table 2**). Data is harmonized by conversion to percentage values. Data was statistically analyzed to study variations, nutrient correlation, correlation with distance from the equator, and climatic zone.

**Table 2:** Country-wise studies on nutritional composition of *M. oleifera* seeds.

S. no.	Location	Moisture (%)	Ash (%)	Crude fat (%)	Cude fiber (%)	Crude protein (%)	Total carbohydrates (%)	References
Nigeria								
1.	Oyo	5.95	3.38	43.6	17.6	43.71	3.36	Sodamade et al., 2017
2.	Wudil	9.08	4.64	19.63	6.06	30.09	26.45	Fagwalawa et al., 2014
3.	Oyo	9.97	3.87	38.67	2.87	35.97	8.67	Olagbemide and Philip, 2014
4.	Iloein	9.6	4.03	14.16	30.64	25.37	–	Annongu et al., 2014
5.	Okigwe Imo State	9.56	8.24	2.6	13.4	17.94	48.26	Mgbemena and Obodo, 2016
6.	Anambra State	12.63	6.22	14.93	7.1	13.92	45.2	Umerah et al., 2019
7.	Oyo	4.7	4.1	45.84	7.73	28.04	10.59	Abiodun et al., 2012
8.	Ife	–	3.2	37.1	2.9	41.9	14.9	Ogunsina et al., 2015
9.	Nigeria (unspecified)	8.4	4.5	13.0	8.6	22.5	43.0	Musa and Njidda, 2021
Ethiopia								
10.	Ethiopia (unspecified)	6.1	–	28.5	–	34.7	–	Stadtlander and Becker, 2017
Malawi								
11.	Central Malawi	2.6	4.6	41.18	4.8	37.86	11.55	Chatepa and Mbewe, 2018
Cameroon								
12.	Cameroon (unspecified)	7.7	5.2	1.7	3.7	58.7	22.3	Saa et al., 2022
Egypt								
13.	Fayoum University	5.3	–	34.0	–	22.6	–	Ebeid et al., 2020
14.	Egypt (unspecified)	–	6.3	36	–	24	33.6	Abdalla et al., 2022
India								
15.	Assam	6.78	3.5	39.12	–	40.34	8.94	Liang et al., 2019
16.	India (unspecified)	4.8	–	37	–	37.5	–	Stadtlander and Becker, 2017
17.	India (unspecified)	3.32	5.73	36.63	–	39.04	15.27	Illingworth et al., 2022
18.	Mysuru	7.8	3.87	13.5	9.2	34.57	28.65	Rajendra et al., 2020
19.	Madurai	4.1	2.5	0.1	5.5	3.1	–	Chelliah et al., 2017
20.	Chennai	5.3	2.3	0.1	4.2	3.8	–	Chelliah et al., 2017
Malaysia								
21.	Malaysia (unspecified)	4.0	6.3	20.1	21.0	26.5	27.5	Abdulkadir et al., 2016
Pakistan								
22.	Faisalabad	6.53	6.61	30.94	7.46	35.26	19.89	Ilyas et al., 2015
Republic of Congo								
23.	Bacongo	5.3	4.2	39.3	–	37.6	13.6	Kimbonguila et al., 2010
Ecuador								
24.	Ecuador (unspecified)	–	3.34	28.73	24.41	28.01	35.32	Coello et al., 2020
Brazil								
25.	Etchojoa, Sonora	–	4.48	41.7	–	38.55	8.45	Guzmán-Maldonado et al., 2020
26.	Acapulco, Guerrero	–	4.31	42.27	–	38.57	5.82	Guzmán-Maldonado et al., 2020
Nicaragua								
27.	Nicaragua (unspecified)	4.9	–	37.4	–	36.7	–	Stadtlander and Becker, 2017

## 3 Statistical analysis

Statistical analysis was performed using R 4.5.0 software in Rstudio (posit). The missing data in both datasets were handled using multiple imputation (m=5) with the mice package in R, employing predictive mean matching (PMM). This method was chosen because it maintains the variability and distribution of numeric variables while allowing robust downstream statistical analyses like correlation and meta-regression (van Buuren and Groothuis-Oudshoorn, 2011). The variables used in the imputation model were seed morphological traits (weight, length and width) and compositional traits (ash, moisture, crude fat, crude protein, crude fiber and total carbohydrates). Convergence diagnostics were not explicitly plotted, but the imputation process was performed under default monitoring and inspected for consistency of generated values.

Multiple imputation was employed to handle missing morphological data for getting trait correlation only. Because the dataset we retrieved was so small and incomplete to draw any correlation without imputation. Other analyses were performed without imputation to eliminate chances of any bias that may arise in small datasets because of PMM. The correlation between variable components was computed using Pearson’s method to identify interacting traits.

## 4 Results

### 4.1 Morphological characteristics of *M. oleifera* seeds

In view of the wide distribution of moringa across the globe, it is expected to have variability. The moringa seeds are globose or oval in shape. These bear a tan to brown colored seed coat with three equidistant peppery wings, which are usually brittle and cream to light brown in color.

**Figure 3:**
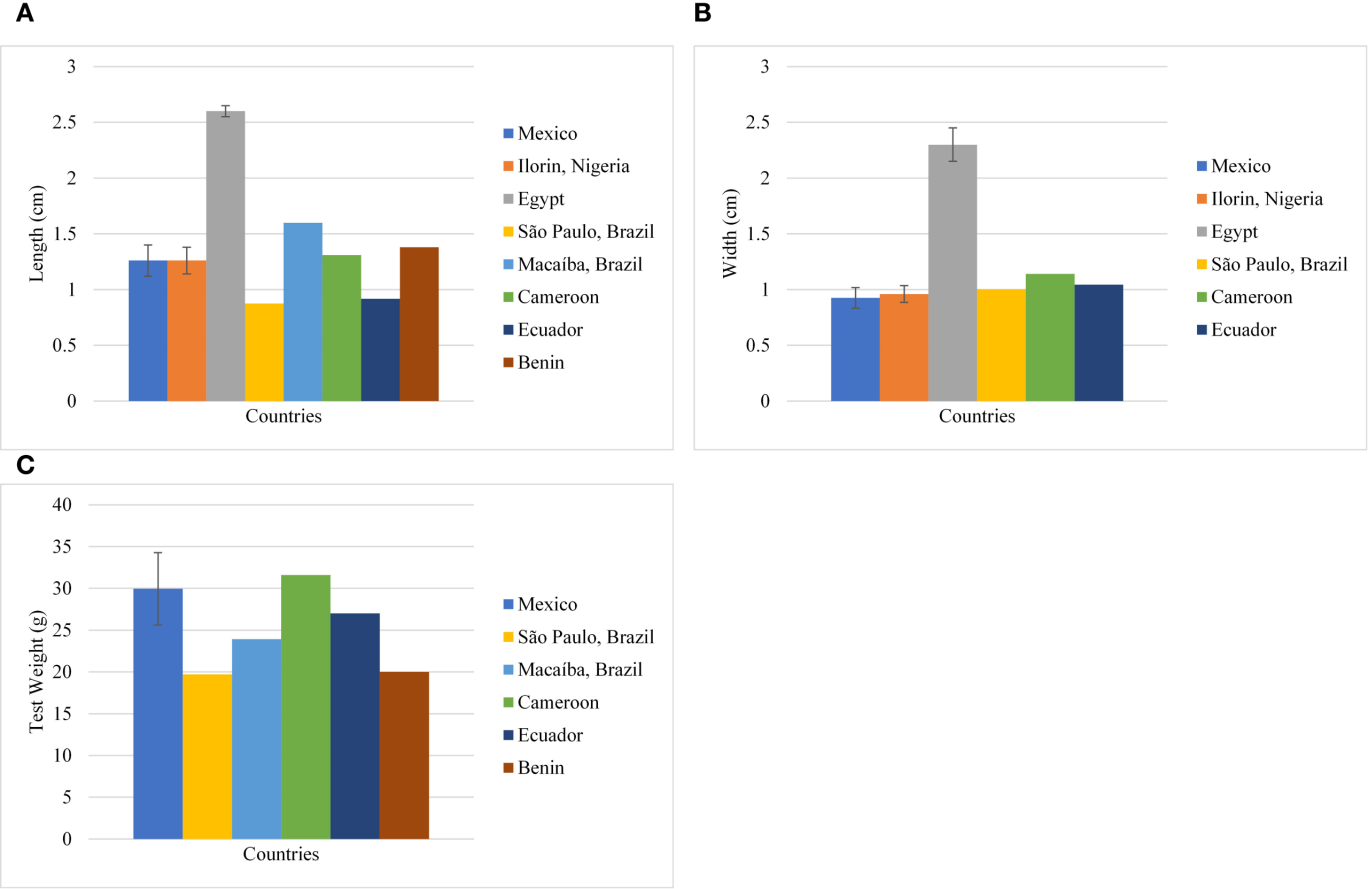
Variability (Mean ± SD) of morphological traits in *M. oleifera* seeds with location. **(A)** seed length (cm) **(B)** width (cm); **(C)** Test weight.

With the limited data available to date, the variability in length, width, and test weight of seeds was calculated. Test weight with coefficient of variance (CV) 19.27%, was moderately variable while seed length and width were highly variable with CV 38.52% and 43.12% respectively (**Table 3**; **Figure 3**). The seed size index, roundness, and elongation were calculated with the available dimensions, and a quantitative measure of the shape was obtained. The quantitative measure predicted a spherical and elliptical shape (**Table 4**), which is in agreement with the qualitative measure in **Table 1**.

**Table 3:** Global variations in width, length and test weight of *M. oleifera* seeds.

Trait	No. of locations	Global mean	Global SD	Global CV (%)	Min mean	Max mean	Range
Width (cm)	6	1.23	0.53	43.17	0.92	2.3	1.37
Length (cm)	8	1.4	0.54	38.52	0.88	2.6	1.73
Test Weight (g)	7	24.83	4.78	19.27	19.7	31.6	11.9

**Table 4:** Seed size index, values of roundness and elongation for *M. oleifera* seeds.

Country	Length (cm)	Width (cm)	Size index (cm^2^)^*^	Roundness^*^	Elongation^*^	Shape^#^
Mexico	1.26	0.925	1.1655	0.734	1.362	Ellipsoidal
Ilorin, Nigeria	1.26	0.96	1.2096	0.762	1.312	Ellipsoidal
Egypt	2.6	2.3	5.98	0.885	1.13	Spherical
São Paulo, Brazil	0.875	1.001	0.875875	1.144	0.874	Spherical
Macaíba, Brazil	1.6		–	–	–	–
Cameroon	1.31	1.14	1.4934	0.87	1.149	Spherical
Ecuador	0.917	1.044	0.957348	1.138	0.878	Spherical
Benin	1.38	–	–	–	–	–

^*^Calculated from length and width data

^#^Shape is estimated from roundness and elongation.

‘-’ Indicate that data is unavailable.

**Figure 4:**
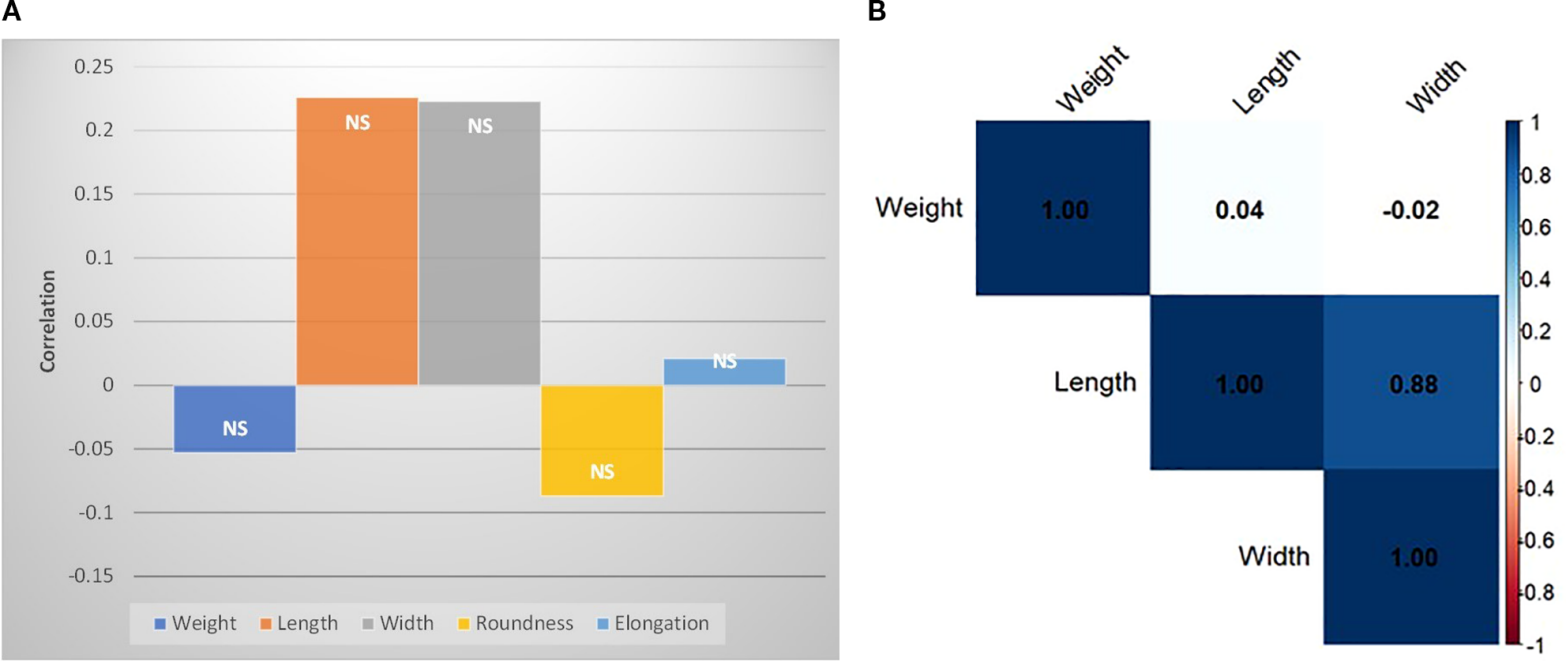
Pearson’s correlation of *M. oleifera* seed morphological traits **(A)** with distance from equator, **(B)** among weight, length and width.

The correlation of the morphological traits (length, width, weight, elongation and roundness) with absolute distance from the equator is statistically non-significant (**Figure 4A**). Although a very high positive correlation (p-value= 0.0008) was found between length and width (**Figure 4B**). Surprisingly, there was no correlation of these traits with test weight.

### 4.2 Nutritional composition of *M. oleifera* seeds

**Figure 5:**
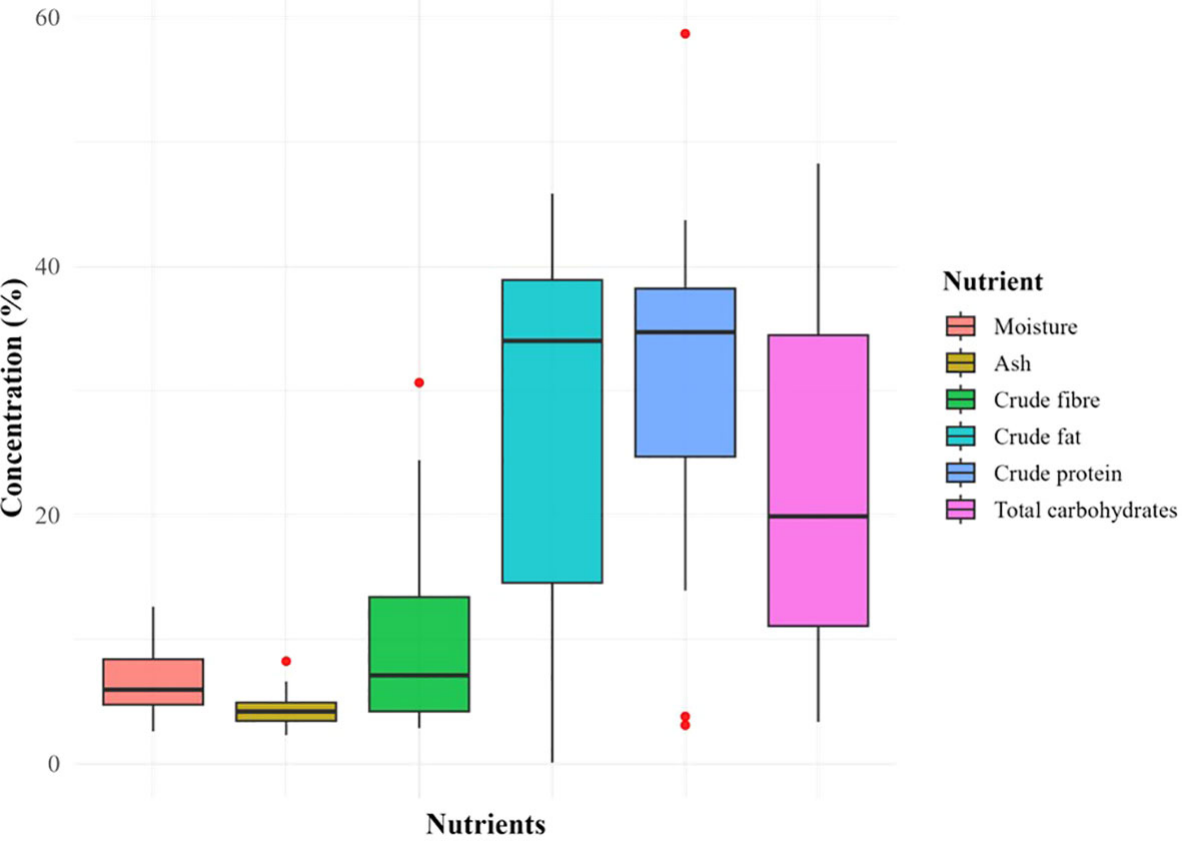
Variability in proximate composition of *M. oleifera* seeds.

In the selected scientific literature, most of the researchers removed hull while seed processing before biochemical analysis. Therefore, the data presented here is for dehulled seeds. Boxplots in **Figure 5** represents central tendency and variability in the nutrient composition of the seeds. The crude fat, total carbohydrates and crude protein were present in abundance with high variability (σ = 14.56, 14.54 and 12.08 respectively) across the globe. Ash and moisture were least abundant and least variable (σ =1.41 and 2.48 respectively) while crude fiber showed intermediate variability (σ = 2.87) as well as abundance. The crude fat showed highest range among the parameters. Some outliers present the possibility of further widening of the range as research progresses and more data is available (**Supplementary File 2**).

### 4.3 Correlation among nutrients in *M. oleifera* seeds

**Figure 6:**
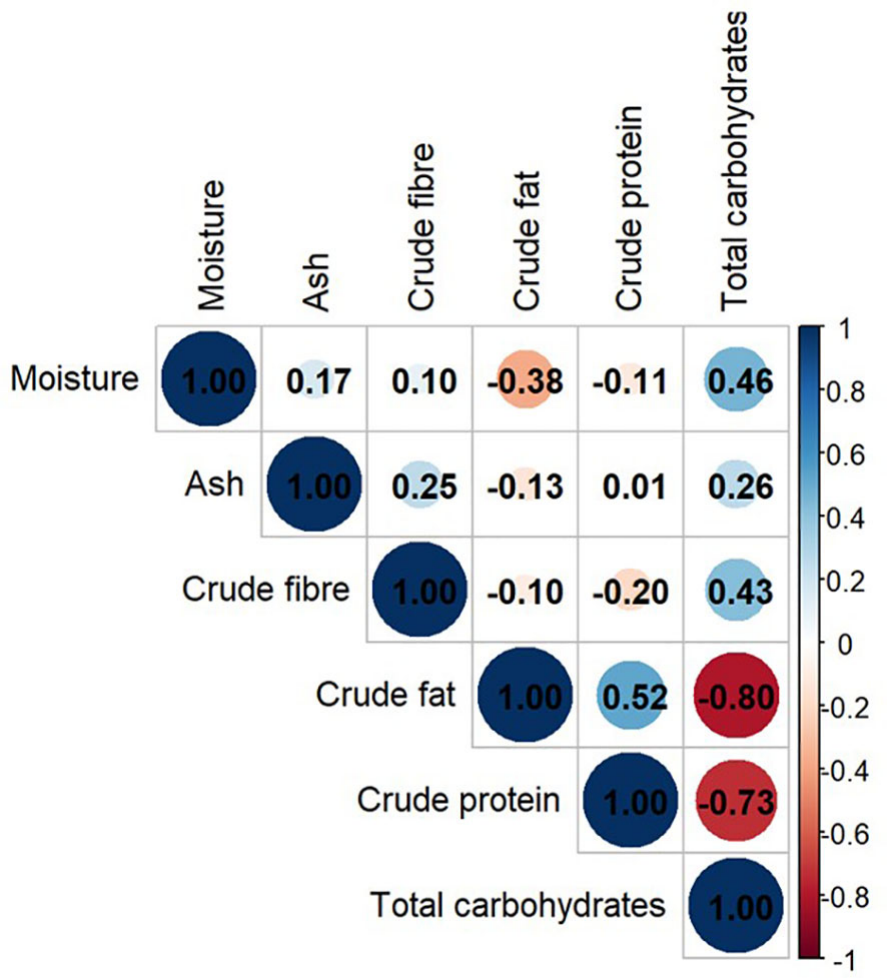
Pearson’s correlation of nutritional components in *M. oleifera* seeds.

In the Pearson’s correlation matrix, total carbohydrates correlated with most nutritional components. It had a correlation with crude fat (r-value = -0.80 and p-value = 0), crude protein (r = -0.73, p-value = 0), crude fiber (r = 0.43, p-value = 0.026) and moisture (r = 0.46, p-value = 0.016) as shown in **Figure 6**. Crude fat and crude protein were moderately positively correlated (r = 0.52, p-value = 0.005). The crude fat and protein had little or no correlation with other nutritional components (**Figure 6**, **Supplementary File 3**).

### 4.4 Correlation of nutritional components with distance from equator

**Figure 7:**
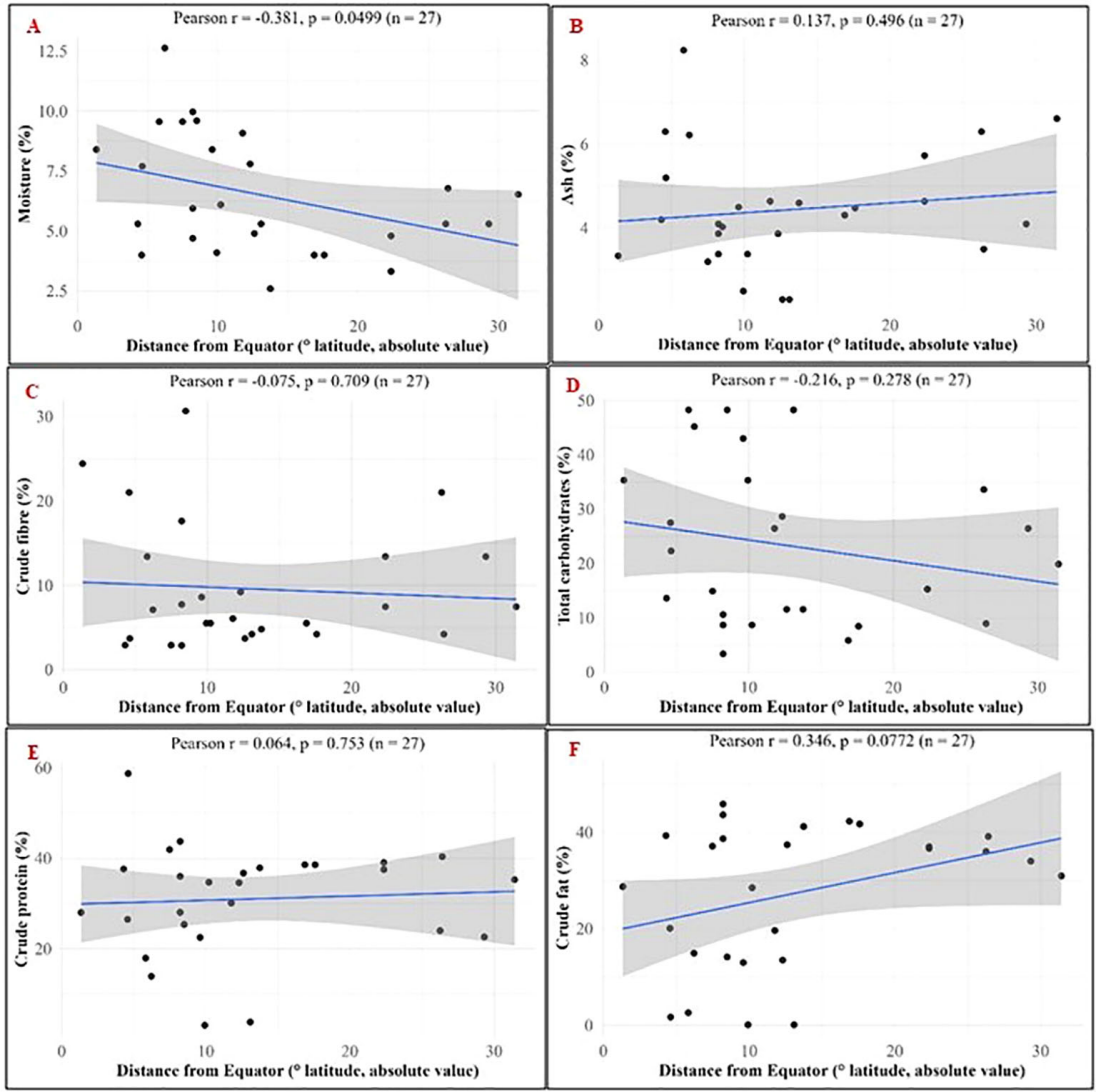
Pearson’s correlation of nutritional components in *M. oleifera* seeds with absolute distance from the equator. **(A)** Moisture; **(B)** Ash; **(C)** Crude fibre; **(D)** Total carbohydrates; **(E)** Crude protein; **(F)** Crude fat.

The correlation of moringa seed nutritional components with the absolute distance from equator was also computed (**Figures 7A–E**). A moderate correlation was found for moisture (r = -0.381, p = 0.0499), crude fat (r = 0.346, p = 0.0772), and total carbohydrates ((r = -0.216, p = 0.278), (**Figures 7A, F, D**). The moisture content decreased with increasing distance of location from the equator. This negative correlation was statistically significant. But, positive correlation of the crude fat and negative correlation of the total carbohydrates with increasing latitude value was not statistically significant.

### 4.5 Influence of climatic zone on nutritional composition of *M. oleifera* seeds

**Figure 8:**
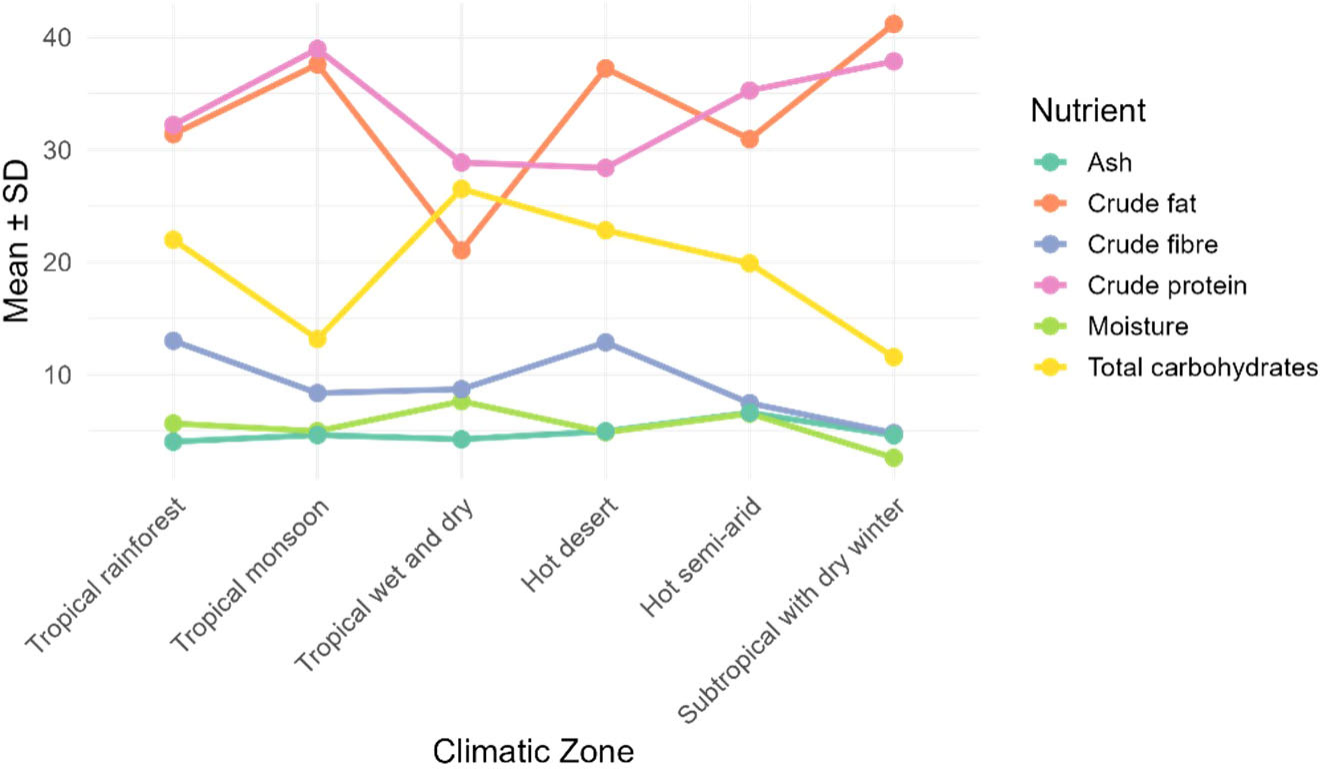
Nutritional trend in *M. oleifera* seeds across climatic zones, moving outwards from the equator and with decreasing mean temperature.

The mean values for each zone were computed and nutrient trend across climatic zone was plotted (**Figure 8**). The climatic zones were arranged in order of increasing temperature and distance from the equator. The ash and moisture varied least and moisture seems to decrease from tropics to subtropics. The crude fiber and total carbohydrates showed clearly a decreasing trend while crude protein and crude fat increased.

**Figure 9:**
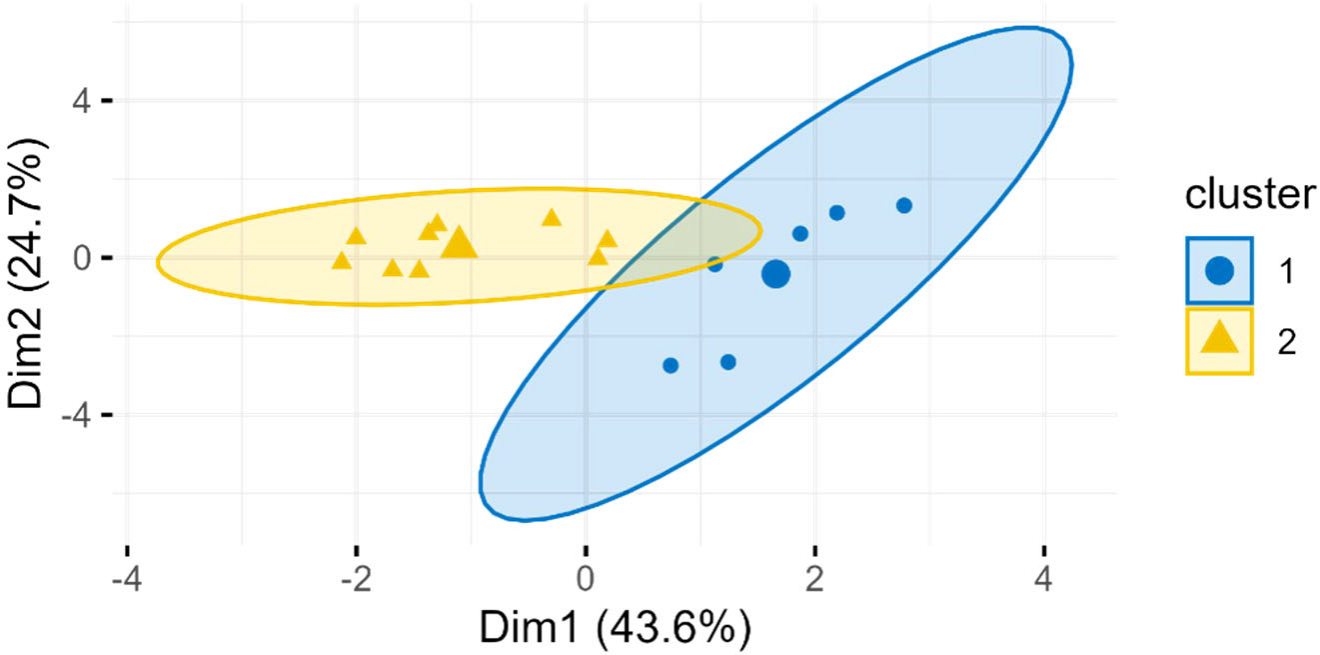
PCA+K-means clustering plot of *M. oleifera* seed proximate nutrients of tropical wet and dry climatic zone.

PCA + K-means clustering was used to divide tropical wet and dry zone data in two probable groups of different seasons. The zone is characterized by distinct wet and dry seasons, the data may represent nutrient profile of both as seen in **Figure 9**.

To evaluate the significance of the preliminary observations of nutrient composition trend across climatic zone analysis of covariance (ANCOVA) was performed by taking climatic zone and latitude as categorical and continuous predictors respectively. Climatic zone had 5 degrees of freedom and latitude had 1 degree of freedom. The F-statistics were low, with corresponding p-values > 0.05 (**Table 5**). The 95% confidence intervals for all predictors overlapped zero, indicating non-significant variation across regions. Neither climatic zone nor latitude had statistically significant effect on any of the nutritional component. The residual variance with 20 degrees of freedom, accounted for most of the variability. Other models can’t be applied here because standard deviation and standard error were not uniformly reported in the selected studies.

**Table 5:** Summary of ANCOVA of nutrients by taking climatic zone and latitude as predictors.

Nutrient	Source of variation	Df	Sum Sq	Mean Sq	F value	Pr(>F)	Significance
Crude protein	Climatic zone	5	350.4	70.08	0.43	0.822	ns
	Latitude	1	183.97	183.97	1.13	0.301	ns
	Residuals	20	3256.93	162.85	–	–	
Crude fat	Climatic zone	5	1472.37	294.47	1.38	0.273	ns
	Latitude	1	3.73	3.73	0.02	0.896	ns
	Residuals	20	4263.32	213.17	–	–	
Crude fiber	Climatic zone	5	122.73	24.55	0.39	0.851	ns
	Latitude	1	40.99	40.99	0.65	0.43	ns
	Residuals	20	1263.76	63.19	–	–	
Total carbohydrates	Climatic zone	5	620	124	0.52	0.759	ns
	Latitude	1	101.25	101.25	0.42	0.522	ns
	Residuals	20	4777.89	238.89	–	–	
Ash	Climatic zone	5	6.85	1.37	0.62	0.687	ns
	Latitude	1	0.44	0.44	0.2	0.662	ns
	Residuals	20	44.23	2.21	–	–	
Moisture	Climatic zone	5	52.31	10.46	1.96	0.129	ns
	Latitude	1	0.65	0.65	0.12	0.731	ns
	Residuals	20	106.83	5.34	–	–	

Ns, non-significant (P > 0.05); Df, degrees of freedom; Sum Sq, sum of squares; Mean Sq, mean square.

## 5 Discussion

*Moringa oleifera* is majorly found in tropical and subtropical regions. The morphological variability exists in the seeds of the crop. Whitish seeds with pale yellow wings are reported in Pakistan (Anwar and Rashid, 2007), which is quite contrasting with other reports included in the study. It may represent a wild cultivar, as the seeds were collected from a forest. The seed weight and size varied with location and no correlation was present between two. This is in contrast to observations of Rafael et al. (2021), where a strong positive correlation was observed in seed morphometric parameters (length and width) with seed weight. This may be due to a lack of available research data and a deficiency of multiple imputation. The Egyptian seeds were exceptionally large in size with length 2.6 cm and width 2.4 cm, but multiple imputations predicted an intermediate weight (**Table 4**). The longer and wider seeds with spherical shape occupy more volume and hence a larger test weight is expected. Seed size and yield are strongly influenced by physiological state of plant or seed which is largely dependent on environmental stressors that may be naturally present in certain locations. Environmental cues activate gene expression of stress responsive genes that result in production of ROS, osmolytes and other defence substances. The stress in seed filling stages compromises on seed storage reserves and hence smaller seeds are produced (Das and Biswas, 2022).

There was no correlation between seed size index and the distance from equator. As the distance from the equator increases, the climate (temperature and rainfall) and nutrient status of soil changes (Hernández-Romero et al., 2024). The climatic and edaphic factors affect physiology of the crops and therefore it is expected as the distance from the equator changes. Absence of correlation with geography may indicates the involvement of genetic factors in determining morphological traits. But, genetically similar seeds of same tree and same pod may differ in size as reported by Noronha et al. (2018). Seeds size varies within a single fruit with respect to position. The largest seed is present at the base of a pod, and the size decreases along the length. Seed size indicates the quantity of reserve nutrients. Larger seeds accumulate higher quantity of carbohydrates, proteins and fats. This necessitates the further investigation on factors affecting seed morphological traits. There is scarcity of quantitative data pertaining to seed color and shape. Different studies reported white to brown colored moringa seeds and seed wings. Seed shape is also found to be variable from spherical to elliptical. Gupta et al. (2022) reported round seed kernel. In the absence of quantitative data, it was not feasible to draw any correlation of shape and color with geographical location. So, we were unable to eliminate chances of subjectivity in the analysis.

Moringa seeds are considered rich source of fat and proteins which is found true in our meta-analysis also. But the nutritional composition was variable across locations. Chelliah et al. (2017); Saa et al. (2022) and Mgbemena and Obodo (2016) reported very low crude fat content in moringa seeds, which was in contrast to other studies. Chelliah et al. (2017) also reported low crude protein content which was also contrasting the fact of moringa seed being a good protein source. Saa et al. (2022) also reported very high crude protein content in moringa seeds. Despite variation, we found a correlation among the nutritional components (**Figure 6**). The crude fat, crude protein and total carbohydrate are food reserves of the seeds. The strong correlation among them represents nutritional trade-off. Ash is an independent component. Low fat and protein content as reported by Chelliah et al. (2017) is consistent with positive correlation between them.

The nutritional composition was correlated with the latitude (absolute distance from equator) and climatic zone. Negative correlation of moisture with latitude was statistically significant. Generally, the atmospheric moisture and average rainfall of a location decreases with increasing distance from the equator and it was reflected in the moisture content of the moringa seeds also. A trend in crude fat, crude protein, crude fiber and total carbohydrate was observed across climatic zones when arranged in order of increasing distance from equator (**Figure 7** and **8**). These observations in nutritional composition were preliminary and further investigated by analysis of variance using climatic zone and latitude as variables. Effect of variables taken into consideration for current study had non-significant impact on nutritional trend. Other climatic factors may also contribute for these observations.

The time of flowering is highly variable in moringa crop. Some varieties flower twice a year while others annually (Dhakad et al., 2023). Hence, fruiting face different seasons. The seasonal variation affects reproductive aspects and fruit yield of moringa (Melo et al., 2020). In dry season, water availability is less, photosynthetic rate and hence, CO_2_ assimilation declines (Rivas et al., 2013). Iqbal and Bhanger (2006) reported the effect of seasonality along with location on *M. oliefera* leaves. The effect of season on oil content and quality has also been reported (Wiltshire et al., 2022). Correlating the seed quality attributes directly with geographical location without considering seasonal influence might present incomplete picture. The tropical wet and dry zone is characterized by distinct wet and dry seasons. The PCA+K-mean clustering plot suggested that the “Tropical Wet & Dry” climatic zone data likely contained two distinct sub-groups, which may represent two different seasons (**Figure 9**). Seasonal variations underscore the importance of temperature and rainfall in determining the nutritional composition of moringa seeds. The seasonal variations may also affect other zones, but sufficient data was not available.

The widening gap between crude fat/crude protein and total carbohydrate food reserves (**Figure 8**), as we moved hotter to cooler climates, was consistent with the decreased photosynthesis and CO_2_ fixation. The availability of sunlight and water is higher in tropical regions (Huntley, 2023). The rate of photosynthesis maximizes to fix CO_2_ and more carbohydrates are synthesized and stored. On the other hand, nutritional status of soil significantly varies from tropics to temperate regions. The soil organic carbon, nitrogen and phosphorus increase with decreasing temperature and rainfall (Hernández-Romero et al., 2024). This is due to higher nutrient cycling with increasing temperature. The availability of nitrogen favors the synthesis of proteins as we move away from the equator. In view of this, not the latitude or the climatic zone directly but indirectly influences the nutritional composition through temperature, rainfall and edaphic factors. Edaphic factors such as soil texture, pH, moisture availability, micronutrient balance, salinity etc. create a microenvironment by interacting with weather and climatic conditions. A thorough study with precise location along with accurate soil and weather data will help decipher the quality determining variables.

The stage of harvesting and seed processing also influence the nutritional status. Some researchers have removed hull before analysis while others have not. Seed hull being protective in nature is particularly rich in fiber and secondary metabolites (Saheed et al., 2019). It also has higher moisture content. Thus, it could affect the overall nutritional composition of the seeds. Environmental stresses not only affect seed morphometry but expression of defence related genes also influence the seed nutritional quality by activating new biochemical pathways or enhancing the metabolic flux through existing pathways. Production of secondary metabolites increases which compete for common metabolic precursors with seed storage reserves. Besides compromising yield for defence and adaptation, these specialized compounds provide medicinal values to the seeds. These generalized observations need further investigation in moringa with climatic variations.

**Figure 10:**
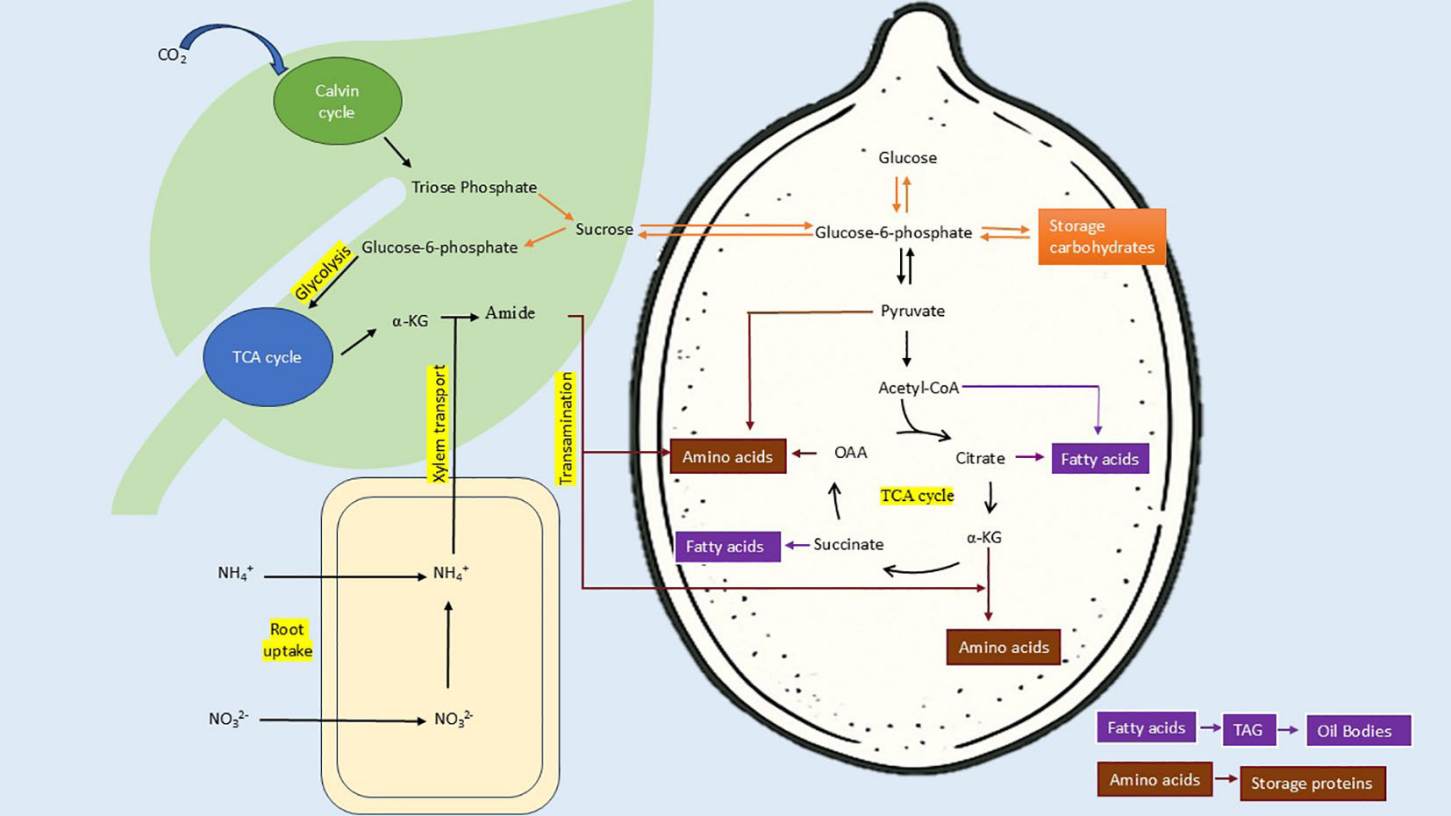
Schematic representation of carbon-nitrogen metabolism and accumulation of food reserves in seeds. Carbon dioxide is fixed in Calvin cycle to produce triose phosphate, which is used to synthesize sucrose for translocation via phloem. Sucrose is translocated to all parts of plant, provide assimilates to glycolysis and TCA cycle. Nitrate fixed in roots is reduced and transported via xylem and used in reductive amination of TCA intermediates via GS-GOGAT or Glutamate dehydrogenase mechanism to produce amides. These amides are translocated to developing seeds to provide amino group. Availability of amides promotes synthesis of amino acids by utilizing TCA intermediates and increases the metabolic flux via pathway. In the absence of amides, sucrose is mobilized to produce storage carbohydrates, which otherwise degraded to Acetyl-CoA and TCA intermediates. This process increases the availability of Acetyl-CoA, Citrate and Succinate for fatty acid synthesis. Fatty acids are incorporated in Triacylglycerols (TAGs) and stored in oil bodies. Amino acids are used for storage protein synthesis. (α-KG, α-Ketoglutarate; OAA, Oxaloacetate; TCA, Tricarboxylic acid; Carbohydrate metabolism, Amino acid metabolism, Fatty acid metabolism) .

In this analysis, a strong negative correlation (r = -0.73, p-value = 0) was observed in the total carbohydrates and the crude protein (**Figure 6**). This relationship can be traced back to carbon-nitrogen metabolism. The carbon-nitrogen metabolic pathways are related to each other (**Figure 10**). The negative correlation between the two is due to diversion of photosynthetic assimilates to nitrogen metabolism with increasing availability of soil nitrogen. CO_2_ fixation is not affected by nitrogen availability (Champigny, 1995). But, Triose phosphate, first stable product of photosynthesis, is partitioned more towards producing reducing equivalents for reduction of nitrate. After nitrogen uptake, a reasonable fraction of carbohydrates is mobilized to provide carbon skeleton for protein biosynthesis (Kaur et al., 2021). Temperature and radiation availability is higher at equator but nitrogen availability is low due to increased nitrogen cycling. With increasing distance from equator average temperature and radiation decreases and hence nitrogen availability increases. Therefore, an overall decrease in CO_2_ fixation with concomitant increase in nitrogen utilization is observed. Thus, negative correlation of carbohydrate and protein is observed with increasing distance from equator.

The positive correlation between crude fat and protein (r =0.52, p = 0.005) is consistent with the observations of Maestri et al. (1998) in soyabean and Babatunde et al. (2022) in different parts of fruits and seeds of large number of plants. But these observations are in contrast to Hymowitz et al. (1972) and Filho et al. (2001). Our observations are also in contrast to observations of Azam et al. (2013) and Hossain et al. (2019) in *Brassica napus*. Oil and protein content are controlled by same SNP locus in Brassica napus (Tang et al., 2019). The positive correlation of the two could be because of overall increase in metabolic flux via Tricarboxylic acid (TCA) cycle with changes in the C:N ratio of soil during seed filling. With decreasing C:N ratio (increased nitrogen availability), sucrose breakdown to provide C-skeleton via TCA cycle for amino acid and protein synthesis (Kaur et al., 2021). Glucose-6-phosphate, phosphoenolpyruvate and pyruvate are breakdown products of translocated sucrose and can be utilized as the carbon skeleton for fatty acid biosynthesis (Kubis et al., 2004) via TCA intermediates (**Figure 10**). Observations of Allen and Young (2013) were in agreement with our findings. C:N ratio affects protein accumulation while maintaining consistent rate dry weight accumulation. Glutamine not only supply carbon to amino acids; it supplies 9 to 19% carbon for fatty acid synthesis. Weigelt et al. (2008) demonstrated that increased N supply, initiates sucrose mobilization via sucrose synthase, glycolysis and TCA cycle to meet the demand of carbon acceptor for amino acid synthesis. This metabolic control is mediated by genetic regulation that sense changes in C:N ratio rather than N alone (Palenchar et al., 2004).

There was a positive correlation between total carbohydrates and moisture content in moringa seeds (r =0.46, p = 0.016). This is again in compliance with the trend in **Figure 8** i.e. higher photosynthesis in higher moisture availability along with the sunlight which is the case at equator, in tropics. As the atmospheric moisture declines, in subtropics, moisture content and total carbohydrates decline together along with crude fibers in moringa seeds. Globally variations are high (**Figure 5**) but the observations of ANCOVA model (**Table 5**), ruled out the direct effect of climatic zone or latitude. Inter zonal variations are lower than the intra-zonal variations. Factors such as temperature, rainfall and soil nutrient status may have influence on the nutritional composition of moringa seeds.

A limitation of this study is that data extraction was conducted primarily by one reviewer, although all numerical values were independently verified for accuracy. Future studies may benefit from duplicate data extraction with inter-rater reliability assessment to further minimize extraction bias. Another limitation of our approach is that morphological and nutritional datasets could not be analytically integrated due to the absence of shared study identifiers. Future research incorporating paired datasets from coordinated sampling could enable correlation or multivariate modelling linking morphology and biochemical composition.

## 6 Conclusion

The spherical to oval shaped moringa seeds varied in color from whitish to brown. similarly, variability was found in the seed dimensions. Also, the current study did not observe any relationship among morphological traits, geographical location and climatic factors. Nutritional composition of moringa seeds was highly variable with highest variance (σ^2^) in crude fat (220.75), followed by total carbohydrate (211.51), crude protein (145.82) and crude fiber (54.90). As a preliminary observation, the tropical climates produce moringa seeds rich in carbohydrates while subtropical climates favor protein and fat accumulation. But statistical model fitting (ANCOVA) revealed that the latitude and the climatic zone are not deciding factors. The nutritional composition trend can be a function of rainfall (atmospheric moisture), temperature, sunlight and soil nutritional status. Further research is required to determine the effect of these climatic factors and involvement of genetic factors, if any.

The present study reports the correlation among nutritional components of moringa seeds. Total carbohydrates correlated negatively with crude fat and crude protein (r-value = -0.80 and 0.73 respectively) but positively with moisture and crude fiber (r = 0.46. and 0.43 respectively). Crude fat and crude protein had moderate positive correlation (r= 0.52) which is in contrast to related crops. These correlations were traced back to carbon-nitrogen metabolism and represented trade-off in seed storage reserves and consistent with the trend along latitudes. Further studies with more robust datasets can investigate the extent of influence of all possible climatic factors on this nutritional trade off in primary and secondary metabolites.

## Data Availability

The original contributions presented in the study are included in the article/**Supplementary Material**. Further inquiries can be directed to the corresponding author/s.
