# Supplementary material for: Geographical variability in morphology and nutritional composition of Moringa oleifera seeds: a meta-analysis
Source: Front Plant Sci. 2026 Feb 9;17:1720005. doi: 10.3389/fpls.2026.1720005 (PMC12926359; doi:10.3389/fpls.2026.1720005)
Supplement: Supplementary file 3 [file Table1.docx]

Legend linking the numeric values in the maps to the Köppen-Geiger classes.

The RGB colors used in Beck et al. (2023) are provided between brackets.

1: Af Tropical, rainforest [0 0 255]

2: Am Tropical, monsoon [0 120 255]

3: Aw Tropical, savannah [70 170 250]

4: BWh Arid, desert, hot [255 0 0]

5: BWk Arid, desert, cold [255 150 150]

6: BSh Arid, steppe, hot [245 165 0]

7: BSk Arid, steppe, cold [255 220 100]

8: Csa Temperate, dry summer, hot summer [255 255 0]

9: Csb Temperate, dry summer, warm summer [200 200 0]

10: Csc Temperate, dry summer, cold summer [150 150 0]

11: Cwa Temperate, dry winter, hot summer [150 255 150]

12: Cwb Temperate, dry winter, warm summer [100 200 100]

13: Cwc Temperate, dry winter, cold summer [50 150 50]

14: Cfa Temperate, no dry season, hot summer [200 255 80]

15: Cfb Temperate, no dry season, warm summer [100 255 80]

16: Cfc Temperate, no dry season, cold summer [50 200 0]

17: Dsa Cold, dry summer, hot summer [255 0 255]

18: Dsb Cold, dry summer, warm summer [200 0 200]

19: Dsc Cold, dry summer, cold summer [150 50 150]

20: Dsd Cold, dry summer, very cold winter [150 100 150]

21: Dwa Cold, dry winter, hot summer [170 175 255]

22: Dwb Cold, dry winter, warm summer [90 120 220]

23: Dwc Cold, dry winter, cold summer [75 80 180]

24: Dwd Cold, dry winter, very cold winter [50 0 135]

25: Dfa Cold, no dry season, hot summer [0 255 255]

26: Dfb Cold, no dry season, warm summer [55 200 255]

27: Dfc Cold, no dry season, cold summer [0 125 125]

28: Dfd Cold, no dry season, very cold winter [0 70 95]

29: ET Polar, tundra [178 178 178]

30: EF Polar, frost [102 102 102]
